# Case Report: Improving gait and fatigue in multiple sclerosis through wearable electrical stimulation: a single-patient study

**DOI:** 10.3389/fresc.2026.1759293

**Published:** 2026-04-10

**Authors:** Serena Filoni, Francesco Romano, Daniela Cardone, Serena Urbano, Domenico Intiso, Raffaello Pellegrino, Emanuele Francesco Russo, David Perpetuini, Arcangelo Merla

**Affiliations:** 1 Unit of Neuro-Rehabilitation, IRCCS “Casa Sollievo della Sofferenza”, San Giovanni Rotondo, Italy; 2 Department of Engineering and Geology, University “G. d'Annunzio” of Chieti-Pescara, Pescara, Italy; 3 Department of Medicine and Surgery, LUM University, Casamassima, Italy; 4 Padre Pio Foundation and Rehabilitation Centers, San Giovanni Rotondo, Italy

**Keywords:** balance rehabilitation, gait, instrumented balance and strength assessments, multiple sclerosis (MS), transcutaneous electrical stimulation (TENS)

## Abstract

Multiple sclerosis (MS) is a chronic disorder of the central nervous system characterized by progressive impairments in gait, balance, coordination, and fatigue. The Exopulse Mollii Suit (EMS) has been recently introduced as a non-invasive method for delivering peripheral surface electrical stimulation to alleviate motor dysfunction in individuals with neurological disorders. This case study evaluated the effects of a 1-Month home-based EMS intervention on functional and biomechanical outcomes in a woman with relapsing-remitting MS. Specifically, a 53-year-old woman, clinically stable and not receiving pharmacological treatment, used the EMS suit every other day for 60-minute sessions over one month. Assessments were conducted at Baseline, Post-Session (after one EMS use), after 1-Month of therapy, and at Follow-Up (one month after therapy cessation). Clinical evaluations included the 6 Min Walk Test (6MWT), 10-Meter Walk Test (10MWT), Timed Up and Go (TUG), and Modified Fatigue Impact Scale (MFIS). Additionally, instrumented balance and strength assessments were performed using the Hunova robotic platform. Progressive improvements were observed in all clinical outcomes: 6MWT distance increased by 28 meters, gait speed improved by 14%, TUG times decreased, and MFIS scores reflected a 30% reduction in fatigue. Balance robotic evaluations showed improvements in postural control, center of pressure metrics, and stabilization times. Notably, improvements were observed after a single session, became more consistent after one month, and were partially sustained at Follow-Up, but a regression toward baseline was observed for some outcomes. Hence, the EMS appears to be a promising home-based intervention for improving gait, balance, and fatigue in MS. However, further studies should be performed to validate these findings in larger cohorts.

## 1 Introduction

Multiple sclerosis (MS) is a chronic autoimmune-mediated disease of the central nervous system characterized by inflammation, demyelination, and axonal damage (1). In MS, immune cells attack the myelin sheath insulating neuronal axons in the brain and spinal cord, leading to focal demyelinated lesions (“plaques”) that impair nerve signal conduction (1). Early in the disease, inflammatory episodes cause blood-brain barrier breakdown and myelin loss; over time, cumulative damage and incomplete remyelination result in gliotic scarring and neurodegeneration (2). Lesions can occur in motor pathways (cortical spinal tracts, spinal cord), cerebellum, and other regions, producing a wide range of neurological deficits (3), and causing disabilities in young adults (4). Specifically, the demyelination of motor neurons disrupts signals to muscles and produce muscle weakness (5). Moreover, spasticity affects up to 80% of MS patients at some point and is associated with impaired ambulation, painful spasms, and risk of contractures (6). Notably, the combination of muscle weakness, spasticity, and sensory loss results in balance and gait impairment. Among the balance and walking difficulties experienced by the MS patients, the ataxia is a spread symptom, manifesting as unsteady or uncoordinated movements due to cerebellar or proprioceptive pathway lesions (7). In advanced cases, cerebellar ataxia combined with spasticity can be severely disabling, leading to wide-based, staggering gait and frequent falls (7). These motor deficits often interact with fatigue, another prevalent MS symptom, to further worsen functional mobility (8). Overall, motor dysfunction, encompassing spasticity, weakness, ataxia, and fatigue, is one of the most disabling aspects of MS and a major contributor to loss of independence (9).

Regarding the MS clinical treatment, a combination of disease-modifying therapies and symptomatic treatments is used to manage the pathology and to address MS-related motor impairments. To this aim, pharmacological treatments for spasticity, including muscle relaxants, are widely employed (10–12). Moreover, in cases of focal spasticity, botulinum toxin injections may be used to relax specific muscles (13). Together with these pharmacological approaches, physical therapy, exercise, and rehabilitation are administered to maintain strength, mobility, and balance. One non-invasive intervention that has gained interest in neurological rehabilitation is transcutaneous electrical nerve stimulation (TENS) (14), which involves delivering low-voltage electrical currents through surface electrodes on the skin (15). In MS, TENS is being explored as a tool to modulate neuromuscular function and improve motor symptoms (16). Growing evidence indicates that beyond pain relief, TENS can influence motor pathways and spasticity (15–17), increasing muscle power output and improving movement function in patients with neurological injuries, while also decreasing spasticity​ (18). Specifically, high-frequency TENS (e.g., −100 Hz) primarily activates large-diameter afferents (A*β* fibers), engaging segmental inhibitory mechanisms that can dampen pain and reflex activity (19, 20), whereas low-frequency stimulation can produce repeated muscle twitching and activate small-diameter afferents, which may induce reciprocal inhibition of overactive motor neurons (19, 20). Repeated TENS sessions may also promote neuroplastic changes, potentially reducing abnormal neural excitability over time (21). Clinical studies on TENS for spasticity and motor performance in MS and other conditions have yielded promising results. For instance, in a pilot trial with MS patients, daily high-frequency TENS (100 Hz, 20 min for 4 weeks) applied to spastic ankle muscles led to significant reductions in muscle spasticity as measured by electromyography (EMG) and the Modified Ashworth Scale (MAS) (22). Although gait speed (Ambulation Index) did not significantly improve in that short intervention, the reduction in hypertonia suggests TENS can relax spastic muscles (22). Notably, some studies have shown a dependence between the effectiveness of TENS and the dosage (16), suggesting that while a brief TENS session may not dramatically alter muscle tone in MS, prolonged or repeated use might yield benefits in terms of muscle relaxation and symptom relief. In this perspective, the possibility to continuously apply TENS at-home could be highly beneficial for the patients. In this perspective, a technological advancement for TENS administration is represented by the Exopulse Mollii Suit (EMS), an innovative full-body garment designed to deliver TENS therapy conveniently to individuals with neurological disorders (21). The suit consists of a jacket and pants integrated with 58 electrodes strategically positioned over major muscle groups. It includes a programmable control unit worn at the waist, which allows therapists or users to adjust stimulation parameters (intensity, frequency, pulse width, timing) to tailor the treatment to the individual (21). The EMS delivers low-level electrical impulses to the body's muscles and sensory nerves; critically, it is typically set up to stimulate antagonist muscle groups to those affected by spasticity. By stimulating antagonists at low frequencies and intensities, the suit harnesses the principle of reciprocal inhibition, the natural reflex whereby activation of one muscle (e.g., the triceps) leads to inhibition of its opposing muscle (the biceps) via spinal interneurons (23, 24), aiming to relax hypertonic muscles and improve range of motion. Additionally, the continuous somatosensory input provided by the suit's electrodes may lead to central neuroplastic changes (25), mitigating spasticity and improving motor control over time (21, 25).

As a wearable neuromodulation system, the EMS offers several practical advantages for therapy. First, the electrode stimulus configuration can be customized to target the specific muscles and impairments of each user, allowing highly individualized treatment programs. Second, because the suit is worn while the person is active, therapy can be integrated with functional tasks; users can move, exercise, or practice daily activities during stimulation sessions. This simultaneous practice of movements with neurostimulation may enhance motor relearning and carryover to real-world function (21). The ability to use the suit at home on a regular schedule (e.g., 1 h every other day) promotes more frequent and consistent intervention than what clinic-based therapies alone can provide.

The EMS has been applied in conditions such as cerebral palsy, stroke, spinal cord injury, and MS. A recent multi-center study evaluated the EMS in 44 patients with MS, cerebral palsy, or stroke over a 4-week period. Notably, after a single 60 min session of wearing the suit, patients showed significant immediate improvements in functional balance and reduced fall risk (as measured by increased Berg Balance Scale scores) (26). After four weeks of regular home use (1 h every other day), further gains were observed, with sustained improvements in mobility and continued reduction in fall risk across all groups (26). In the MS subset, this translated to statistically significant enhancements in balance, overall mobility, and quality of life measures compared to baseline (26). These results align with prior smaller studies. For example, in a pilot with MS and other spasticity disorders, participants reported feeling muscle relaxation and improved ease of movement while using the suit, with some noting better balance and less fatigue during daily activities. Notably, patient acceptance of the EMS has generally been positive; users describe feeling safer during movement and experience a sense of “looseness” in previously tense limbs, with minimal discomfort from the stimulation (27).

The effectiveness of the EMS-based therapy can be assessed through clinical tests and platforms for balance and rehabilitation. An innovative development in balance-assessment platforms is the Hunova Robotic Platform for balance and gait rehabilitation developed by Movendo Technology (Genoa, Italy) that provides interactive assessment and training of balance, posture, and lower-limb motor function. The system consists of two independently actuated, sensorized platforms, one on which the patient stands (or places their feet) and one on which they sit (a seated back/support platform), each with two degrees of freedom (allowing tilting forward-backward and left-right) (28). This configuration enables a wide range of exercises in both standing and seated positions, targeting the ankles, knees, hips, and core/trunk. In addition, an integrated trunk accelerometer/sensor is worn by the patient at the sternum to record upper-body sway. Hunova thus enables a comprehensive evaluation of balance under static and dynamic conditions, and it offers greater versatility than traditional static posturography (28). The device's software can apply controlled perturbations or tilts to challenge the patient's balance, or resistive forces to assist/oppose movements, in a highly programmable manner. In essence, Hunova serves as both an objective assessment tool, quantifying balance parameters, postural sway, and limits of stability, and an active training device that guides patients through therapeutic exercises with feedback. A computer interface provides visual and audio feedback to the user during tasks (for example, games or targets that encourage the user to shift weight appropriately), making therapy engaging and motivating (29).

Studies have validated Hunova's assessments against standard balance tests. In people with MS, dynamic posturography measures on Hunova (e.g., center-of-pressure sway during various stance conditions) showed strong correlation with the traditional Sensory Organization Test, confirming Hunova as a valid device for quantifying balance deficits in MS (29). Notably, the employment of Hunova in MS is expanding, with studies suggesting it can detect balance improvements over time and potentially enhance them through robotic-assisted exercise (29).

The present study aims to evaluate the effects of the EMS on balance, gait, and fatigue in a patient with MS. In this single-case study design, a comprehensive motor assessment was conducted after one treatment, after one month of treatment, and one month post-treatment. Objective outcomes of balance, gait, and fatigue were evaluated in each session in order to provide insight into the time course of effects, distinguishing immediate impacts from longer-term effects.

## 2 Materials and methods

### 2.1 The participant

A single female patient (53 years old) with a confirmed diagnosis of MS was recruited for this case study. At enrollment, her neurological disability was mild-to-moderate, with an Expanded Disability Status Scale (EDSS) score of 3.0. This score reflects the presence of moderate disability in one functional system (or mild disability in several systems) without significant impairment of ambulation. The patient's MS was in a stable phase both clinically and radiologically at baseline, where baseline indicates the condition of the patient before the beginning of the rehabilitation process. Moreover, she had experienced no recent relapses and no new lesions on recent magnetic resonance imaging (MRI), indicating a stable disease course. Importantly, the patient has never undergone specific immunological therapy for demyelinating diseases during her clinical history; she was not receiving any disease-modifying therapy or other pharmacological treatments at the time of the study, which minimized confounding influences on motor function.

The patient's primary impairments included difficulty with gait (e.g., reduced foot clearance and mild ataxic features), poor coordination (especially in lower limbs), impaired balance, and pronounced fatigue during daily activities. These deficits were consistent with her EDSS level and were the target of the intervention. The patient was evaluated and monitored in the context of a neurology outpatient unit, with all assessments performed by experienced neurologists and physiatrists. Prior to starting the intervention, the patient provided informed consent and underwent a comprehensive baseline evaluation (T0) to document her gait, balance, and fatigue levels. Given her stable condition and lack of ongoing treatments, she was deemed an appropriate candidate to trial the EMS intervention, hypothesizing that any functional improvements could be attributed to the stimulation protocol rather than spontaneous recovery or medication effects.

### 2.2 The intervention: exopulse stimulation

The intervention consisted of home-based neuromuscular stimulation using the (Ottobock AB, Sweden), a full-body garment with 58 embedded electrodes designed to deliver low-level electrical stimulation to muscles (30). In preparation for the intervention, a training session was conducted at the neurology unit for the patient and her caregiver. During this session, a clinical therapist provided detailed instructions on how to don the EMS, operate its control unit, adjust settings, and monitor for any adverse sensations. The caregiver was included to ensure correct home use and safety throughout the study period. The patient used the EMS at home for a total of one month. Stimulation sessions were scheduled every other day (approximately 3–4 sessions per week) with each session lasting 60 min. This regimen was chosen based on prior evidence of feasibility and efficacy of the in neurological populations (30). All sessions were performed with the patient in a comfortable seated or lying position initially, and she was encouraged to perform light active movements (such as standing or walking exercises) during stimulation as tolerated, to integrate the neuromuscular facilitation into functional activity. The stimulation frequency was set to the suit's standard low frequency (approximately 20 Hz), which has been associated with inhibitory and modulatory neuromuscular effects in spastic paresis (30). No other rehabilitation therapies were added or changed during this month, so the EMS was the sole intervention.

**Figure 1:**
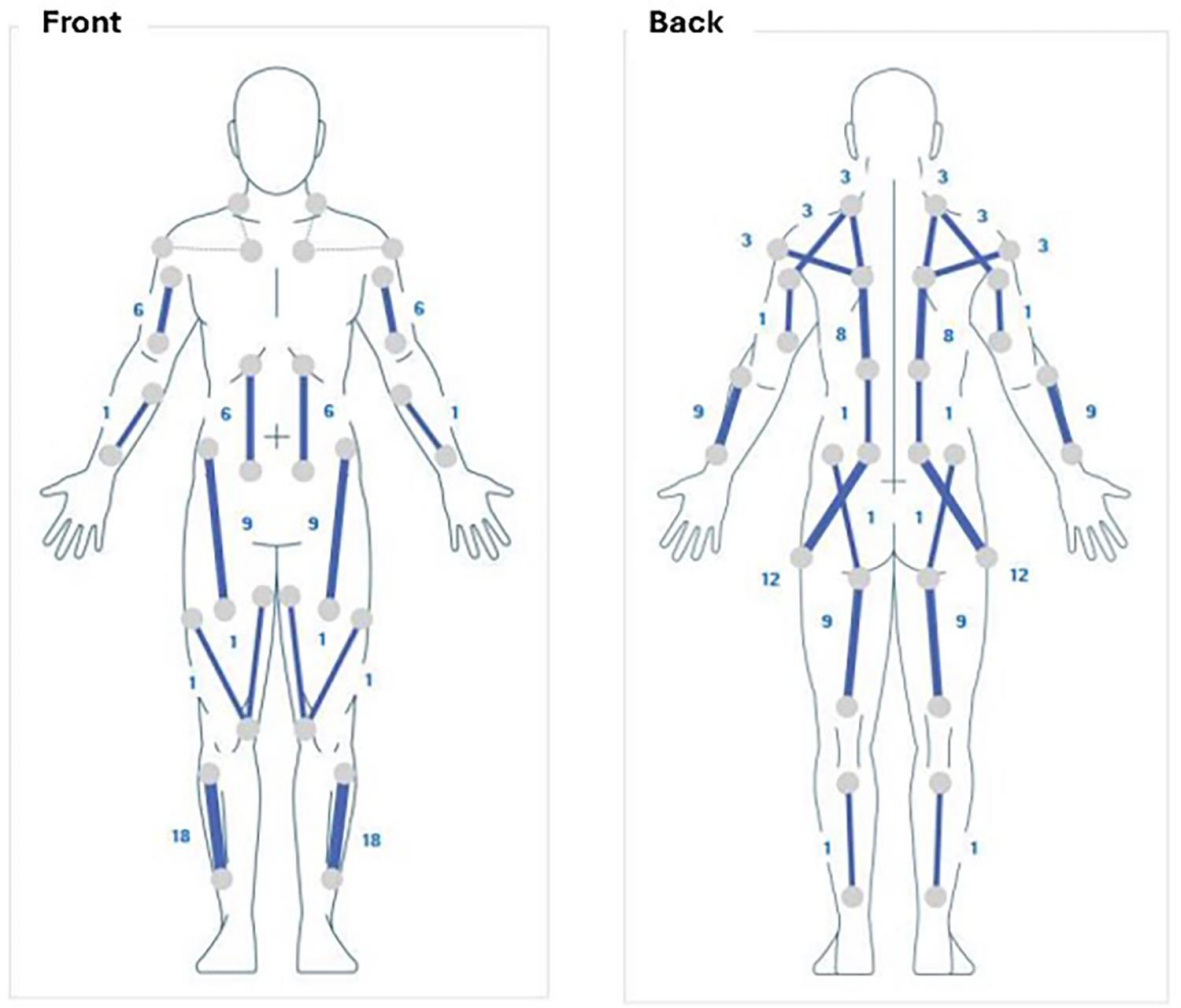
Electrode placement and stimulation pattern of the whole-body EMS.

The electrical stimulation parameters were carefully individualized by the clinical team at T0. The EMS's software allows tailoring of pulse width and intensity for each muscle group. We programmed the device based on the patient's diagnosis and impairment profile, taking into account her age (to ensure comfort), muscle mass (larger muscles requiring slightly higher pulse widths), and symptom severity (areas with greater spasticity or weakness received stronger stimulation). In practice, larger weight-bearing muscles in the legs were assigned longer pulse widths (within the device's typical range of 45–120 µs for lower limbs), whereas smaller upper-limb muscles were set at shorter pulse widths (around 40–90 µs range) (30). Stimulus amplitude (voltage) was kept low (sufficient to elicit sensory response and mild muscle activation but well below pain threshold, max 20 V) (30). This approach ensured the stimulation was strong enough to engage neuromuscular responses in targeted areas without causing discomfort or overt muscle fatigue. Table 1 and Figure 1 summarizes the key muscle groups targeted and their stimulation parameters.

**Table 1:** Targeted muscle groups and stimulation parameters (pulse width) used for the EMS-based treatment for the case patient. Each muscle's stimulation was adjusted based on her individual characteristics. (µs = microseconds).

Body Region	Muscle Group (Target)	Pulse Width (µs)	Stimulation Purpose
Lower limbs	Tibialis anterior (bilateral)	80 µs	Dorsiflexors—facilitate foot lift and gait swing (reciprocal inhibition of calf muscles)
Lower limbs	Quadriceps femoris (bilateral)	100 µs	Knee extensors—improve stance stability and counter flexor spasticity in knees
Lower limbs	Hamstrings (bilateral)	80 µs	Knee flexors—facilitate knee flexion in swing, relax tight quadriceps if present
Lower limbs	Hip flexors (iliopsoas, bilateral)	85 µs	Hip flexors—assist leg swing initiation and improve gait cadence
Trunk	Paraspinal back extensors	60 µs	Postural muscles—enhance trunk upright control and balance
Trunk	Abdominal core muscles	60 µs	Core stabilizers—support balance and posture (mild activation)
Upper limbs	Wrist and finger extensors (bilateral)	50 µs	Forearm extensors—mild stimulation to upper limbs (no major deficit; mainly for generalized neuromodulation)
Other segments	Unaffected muscle groups (various)	25 µs (baseline)	Generalized light stimulation for relaxation on muscles without specific deficits

As shown above, the primary focus was on the lower limb muscle groups critical for ambulation (ankle dorsiflexors, knee extensors/flexors, and hip flexors) as well as trunk postural muscles. These were assigned higher pulse widths to provide functional activation (e.g., facilitating ankle dorsiflexion via tibialis anterior stimulation to mitigate foot-drop and improve gait). In contrast, muscle groups where the patient did not exhibit impairment (“unaffected segments”, such as much of the upper limbs) were still stimulated but only with a very low pulse width (−25 µs). This generalized low-level stimulation was included to promote relaxation and a systemic neuromodulatory effect without causing any significant muscle contraction in those areas. Throughout the 1-Month period, the patient and caregiver kept a log of EMS usage to track session completion and any issues. The patient reported full compliance with the every-other-day schedule, and no adverse events or skin irritations were noted. After the 1-month intervention phase was completed (T2 evaluation), the patient then went through a no-stimulation washout period until the follow-up assessment (T3), allowing to observe whether any gains persisted after ceasing EMS use.

### 2.3 The clinical scales employed

To evaluate the effects of the intervention on the patient's functional motor abilities, a battery of standard clinical outcome measures was employed. These assessments were chosen to quantify gait capacity, speed, balance/mobility, and fatigue—reflecting the main domains of impairment for this patient. All clinical tests were administered at four time points: Baseline (T0) prior to any stimulation, Post-1st Session (T1) immediately after the first 60-minute EMS session (to gauge acute effects), Post-1 Month (T2) at the conclusion of the 1-month home EMS program, and Follow-Up (T3) one month after completing the intervention (to assess retention of effects). The specific scales and tests used were:

- 6 Min Walk Test (6MWT): a measure of functional walking endurance. The patient had to walk back and forth along a 30-meter corridor for 6 min, covering as much distance as possible at a safe, self-determined speed. The total distance walked (in meters) in 6 min was recorded. The 6MWT has been validated in MS populations as a test of walking capacity/endurance (31, 32). A greater distance indicates better functional walking ability, and decreases in distance over time can indicate fatigue.
- 10-Meter Walk Test (10MWT): a measure of gait speed over a short distance. The patient walked a 10-meter straight path at her typical comfortable walking speed (using no assistive device). Timing with a stopwatch began when the first foot crossed the 0 m mark and stopped at the 10 m mark; the test was performed twice and the average time (and corresponding speed in m/s) was calculated. The 10MWT is commonly used in MS and other neurological conditions to quantify walking speed in a controlled setting (33). An increase in gait speed after the intervention would suggest improved neuromuscular control or confidence in walking.
- Timed Up and Go (TUG) Test: a test of functional mobility, dynamic balance, and fall risk. The patient began seated in a chair, then stood up, walked 3 meters, turned around, walked back, and sat down, all “as quickly and safely as possible.” The time to complete this sequence was recorded in seconds. The TUG integrates multiple components of mobility—sit-to-stand transfer, walking, turning, and sitting—and thus is a sensitive measure of overall motor function. It is reliable in persons with MS and correlates strongly with EDSS and with risk of falls (31). By comparing the patient's times across sessions, any improvements in agility and balance were identified (a shorter TUG time indicates better functional mobility).
- Modified Fatigue Impact Scale (MFIS): a patient-reported questionnaire to assess the perceived impact of fatigue on physical, cognitive, and psychosocial functioning. The MFIS is part of the MS Quality of Life inventory and is validated for capturing MS-related fatigue (34). It consists of 21 items (scored on a 0–4 scale) that evaluate how fatigue has affected the patient's activities and thinking in the past weeks. The MFIS was administered at each time point, and calculated the total score (0–84, with higher scores meaning greater fatigue impact). This allowed us to document any changes in fatigue levels that the patient attributed to the stimulation. Fatigue is a critical symptom in MS and was a prominent complaint for this patient; thus, MFIS provided a quantitative gauge of whether the intervention conferred any subjective relief from fatigue in daily life.

Each clinical assessment was conducted by the same evaluator at all time points to ensure consistency. Furthermore, the assessments were always carried out at the same time of day to minimize daily fluctuations in some symptoms such as fatigue. Standardized instructions and scoring protocols were followed for all tests. At baseline (T0), the patient performed the tests without having experienced the EMS, establishing her reference performance. The post-session assessment (T1) was done approximately 30 min after completing the first EMS session on Day 1; this immediate test aimed to capture any acute, transient effects of a single stimulation (for example, temporary changes in muscle tone or alertness). After one month of regular EMS use, the same tests were repeated at T2, allowing for observing cumulative or training effects of the intervention. Finally, the follow-up (T3) evaluation took place one month after the end of EMS usage. During the intervening month (T2 to T3), the patient did not use or undergo new treatments, so T3 provided insight into the durability of any improvements once stimulation was withdrawn. Taken together, this timeline of assessments (Baseline, Post-acute, Post-1 Month, Follow-up) enabled differentiation of short-term vs. longer-term effects of the EMS on clinical outcomes.

### 2.4 Hunova-Based evaluations

In addition to clinical scales, instrumented evaluations using the Hunova® robotic rehabilitation platform were employed to quantitatively measure the patient's balance and motor performance. The Hunova's assessment protocols were used to capture detailed biomechanical data that complement the clinical scales, providing a sensitive gauge of balance and postural control changes over time. The patient underwent a series of Hunova assessments at the same four time points (T0, T1, T2, T3) immediately following the clinical scale tests at each visit. The key Hunova-based evaluations performed were as follows:

- Dynamic Balance: To evaluate reactive balance and the patient's ability to regain stability after disturbances, Hunova's perturbation assessment modules were used. In these tests, the platform actively moved under the patient's feet in controlled patterns (e.g., toes-up, toes-down, or lateral tilt), and the patient's responses were measured. The percentage of adaptation after each perturbation (in %) was measured. Higher trunk oscillation and longer stabilization times indicate poorer dynamic balance.
- 30 s Sit-to-Stand: Using Hunova's instrumented seat, the patient's sit-to-stand performance was evaluated. In this test, the patient started seated on the Hunova chair (which contains force sensors and can record weight distribution). On command, she stood up to a fully upright position. The number of repetition of the stand-up movements in 30 s is recorded. Depending on the number of repetitions performed, this assessment allows for the evaluation of lower limb strength and the patient's endurance.
- Squat Exercise Assessment: it was used to assess dynamic strength and symmetry in a functional movement. The patient was instructed to perform a series of ten squats (from standing to semi-squat and back to standing) at a self-selected pace while standing on the platform. The device's sensors measured the percentage of body weight borne by each leg during the squatting motion, as well as the time durations of the descent and ascent phases for each squat. This test was used to detect improvements in lower-extremity coordination and endurance.

**Figure 2:**
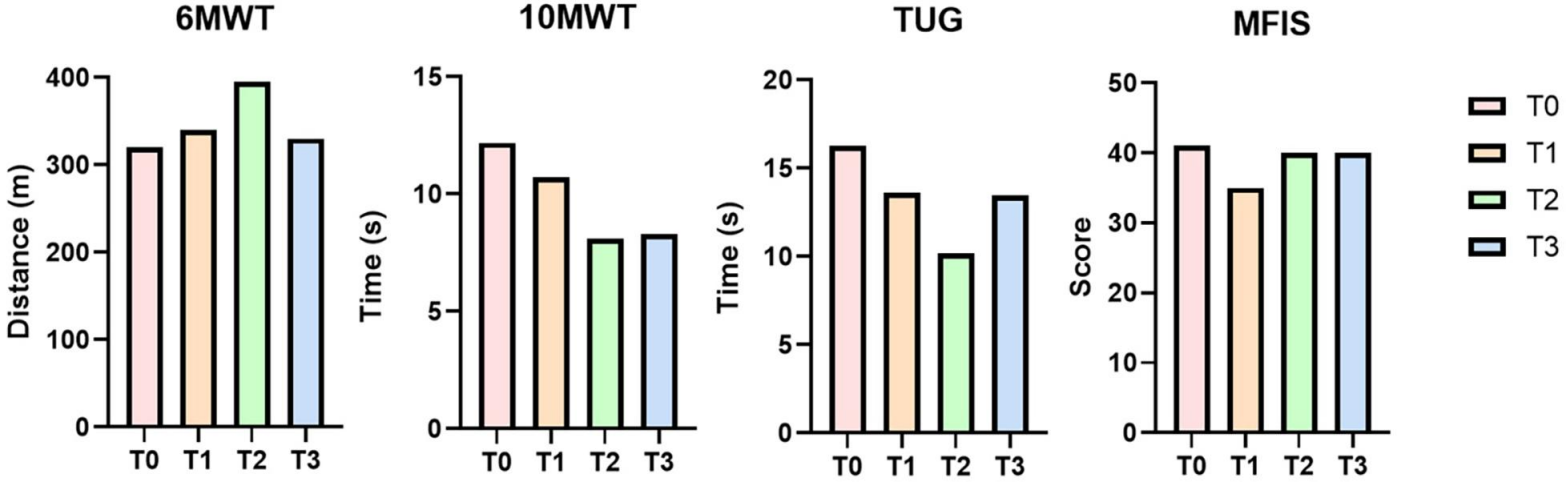
Participant performing a assessment session on the hunova robotic platform.

All Hunova-based evaluations were performed in a safe, controlled manner with the evaluator standing nearby for support if needed. The device's software automatically calculated the various parameters for each test trial. Key outcomes from the Hunova [e.g., Center of Pressure (CoP) sway metrics, trunk sway, reaction times, torque values] were tracked across T0, T1, T2, T3, parallel to clinical outcomes. Figure 2 depicts the participant during the session with the Hunova platform.

## 3 Results

### 3.1 Clinical outcomes

The clinical outcomes demonstrate a consistent trend of functional gains in gait, mobility, and fatigue following use of the EMS, with both immediate and longer-lasting effects. In detail:

- 6MWT: The patient showed a progressive improvement in walking endurance over time. From a baseline distance of 320 m, the distance increased to 340 m after the first EMS session, to 395 m at 1-Month, and slightly declined to 330 m at Follow-Up.
- 10MWT: Walking speed also improved over the study period. The patient's average time to complete 10 meters decreased from 12.18 s at Baseline (0.82 m/s) to 10.73 s Post-Session (0.93 m/s), 8.1 s at 1-Month (1.23 m/s), and 8.3 s at Follow-Up (1.20 m/s).
- TUG: Timed Up and Go scores improved from 16.26 s at Baseline to 13.61 s Post-Session, 10.16 s seconds at 1-Month, and 13.45 s at Follow-Up.
- MFIS: Self-reported fatigue, as measured by the Modified Fatigue Impact Scale, decreased notably. The total score dropped from 41 at Baseline to 35 at 1-Month, with a modest increase to 40 at Follow-Up.

**Figure 3:**
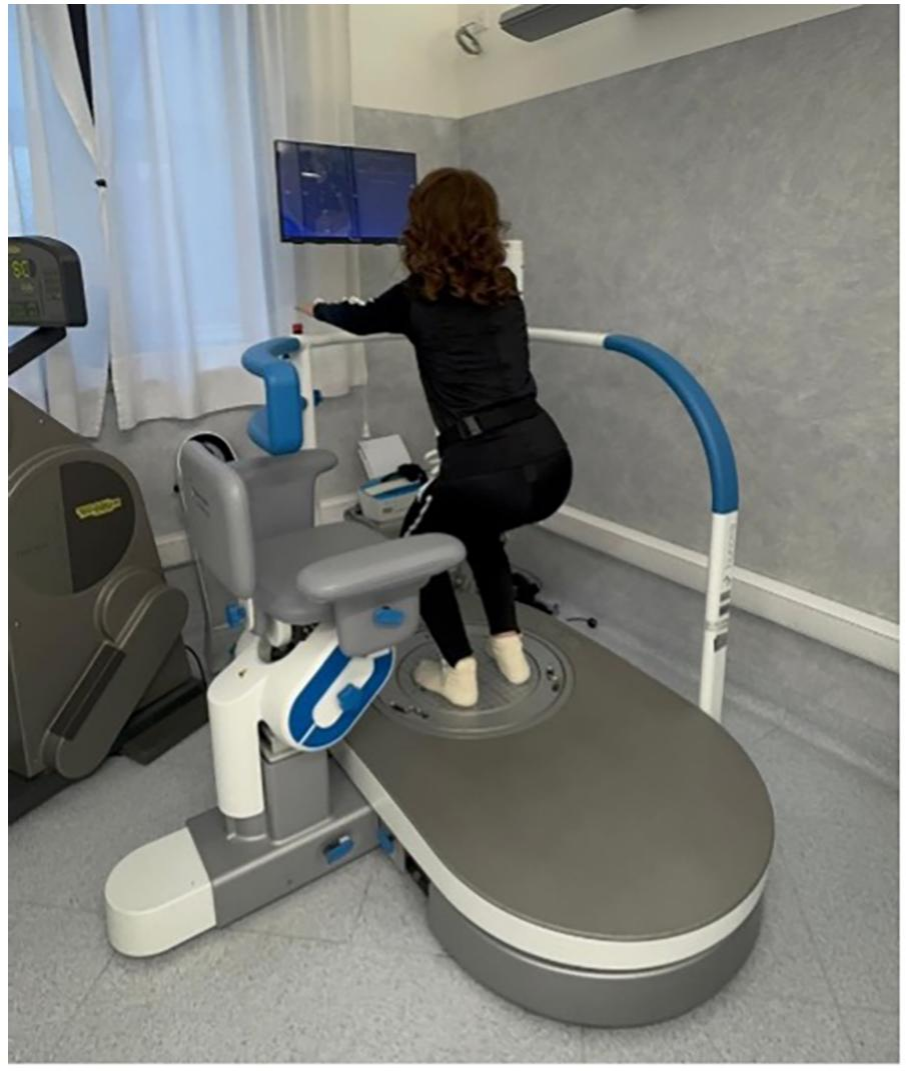
Clinical outcomes at the four time points (T0–T3) for 6MWT distance (m), 10MWT time (s), TUG time (s), and MFIS score.

The results are summarized in Figure 3 and the patient's progress have been compared against established benchmarks and minimal clinically important differences (MCIDs) in MS, as reported in Table 2.

**Table 2:** Patient's progress compared to established benchmarks and minimal clinically important differences (MCIDs) for the clinical measures for MS.

Measure	Baseline (T0)	1-Month (T2)	Change	MCID/Benchmarks in MS
6MWT	320 m	395 m	+75 m	An increase of −20% or 30+ meters is typically considered clinically meaningful.
10MWT	0.82 m/s	1.23 m/s	+0.41 m/s	A change of 0.1 to 0.2 m/s is often cited as the MCID in neurological gait.
TUG	16.26 s	10.16 s	−6.10 s	Times >12 s are often associated with high fall risk in MS.
MFIS	41	35	−6 points	Reflects a −15% reduction in perceived fatigue impact.

### 3.2 Hunova platform-based assessment

Concerning the number of repetitions of the sit-to-stand task in 30 s increased over the study period, from 10 at Baseline to 11 Post-Session, 13 at 1-Month, and 11 at Follow-Up.

Regarding the dynamic balance and perturbance tests, the patient's response to forward perturbation (platform tilt forward) improved markedly after the initial negative response at Baseline (−95.54%), which may reflect an inappropriate or compensatory reaction. Notably, this metric reflects the patient's ability to minimize postural displacement (trunk sway) in response to a sudden platform tilt. The system compares the trunk oscillation measured during the perturbation to a reference of stability A negative adaptation percentage indicates that the patient's trunk moved significantly more than expected or in a direction that worsened the instability. In MS, this often manifests as a stiffened posture that overshoots the corrective movement, or a delayed reaction that fails to arrest the forward momentum created by the tilt. This shifted to a positive adaptation at Post-Session (24.37%) and 1-Month (54.78%), suggesting improved anticipatory control. At Follow-Up, the adaptation decreased to 29.67%, possibly due to the lack of ongoing stimulation.

Concerning the CoP excursion, the maximum forward CoP increased from 6.40 cm at Baseline to 8.16 cm Post-Session, suggesting enhanced capacity for anterior weight shift. Values remained stable through 1-Month (7.12 cm) and Follow-Up (6.79 cm). The Backward CoP increased from 5.90 cm to 7.08 cm at Post-Session, then stabilized at 6.88 cm after one month and to 5.59 cm at Follow-up. Moreover, the total postural sway, showed a decrease from 5.5 cm to 3.78 cm at Post-Session, then decreased to 2.92 cm after one month and increased to 4.99 cm at follow-up, whereas the CoP Ellipse Area decreased from 263.89 cm² to 105.07 cm² post session, to 73.11 cm² after one month and increased to 218.67 cm² at follow-up.

Regarding the trunk oscillation, the AP sway improved from 12.05° at Baseline to 5.40° at Post-Session, 5.20° at 1-Month, and 5.17° at Follow-Up. The ML Sway followed a similar trend: 1.17°, 1.07°, 2.14°, and 0.93°. Reduced trunk oscillation reflects better postural control and core engagement. The patient's ability to regain balance after platform perturbations (e.g., sudden shifts) improved steadily: from 0.78 s at Baseline, to 0.65 s at Post-Session, 0.78 s at 1-Month, and 0.53 s at Follow-Up. This reduction in stabilization time indicates a faster and more efficient balance recovery mechanism.

**Figure 4:**
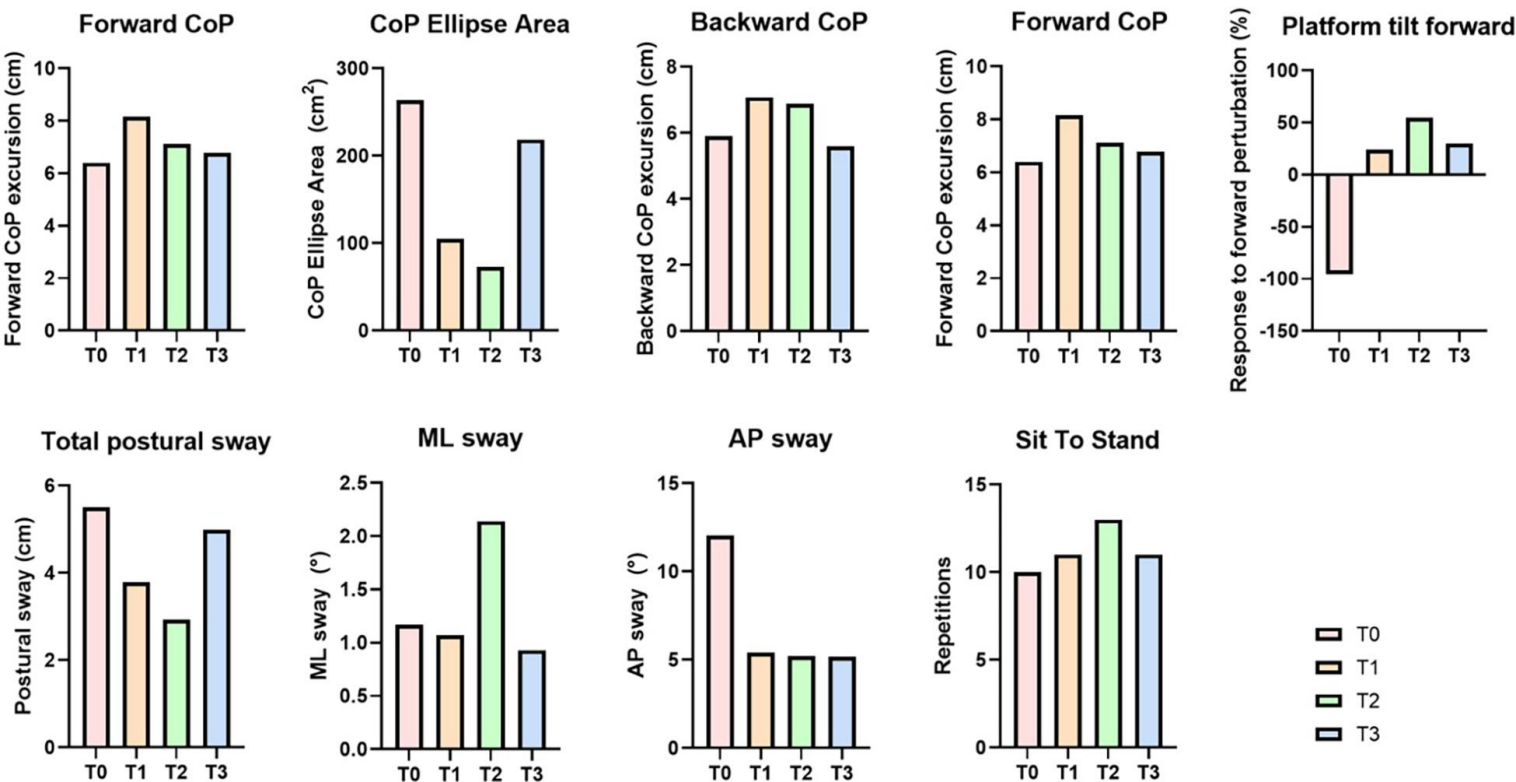
Instrumented hunova outcomes at T0–T3, including CoP excursion and ellipse area, total, ML and AP sway, response to perturbations, platform tilt performance, and sit-to-stand repetitions.

The results are reported in Figure 4.

## 4 Discussion

This case study evaluated the effects of a 1-month home-based intervention using the EMS in a 53-year-old woman with relapsing-remitting MS, classified as EDSS 3.0. The patient used the EMS suit every other day for 60 min across four weeks. Outcome measures were collected at four time points: Baseline, Post-Session (immediately after the first session), 1-Month (after the complete intervention), and Follow-Up (one month after treatment cessation). Clinical and robotic assessments (via the Hunova platform) were employed to measure functional and sensorimotor changes in balance, gait, fatigue, and postural control.

The study revealed significant improvements in walking endurance (6MWT), speed (10MWT), mobility (TUG), and fatigue (MFIS). Parallel improvements in biomechanical metrics, such as reduced trunk sway, enhanced CoP control, faster stabilization times, and increased torque, suggest that EMS induced measurable neuromuscular adaptations. These effects were partially sustained at follow-up, indicating that the benefits were not solely transient. However, it is worth highlighting that a regression toward baseline was observed for some outcomes (i.e., MFIS and postural sway).

The structure of the study allowed a unique opportunity to differentiate between the immediate effects (Post-Session), the cumulative benefits (1-Month), and the sustainability of changes (Follow-Up). Improvements after a single session were evident in several clinical parameters and trunk sway metrics, suggesting that acute neuromodulation, possibly mediated by reciprocal inhibition and afferent activation, can rapidly influence motor control (35).

More pronounced effects emerged after one month of repeated use. The 1-Month gains reflected a cumulative effect of repeated EMS sessions, which may have contributed to plastic changes in motor networks and improved coordination (36). While some decline was observed at Follow-Up, notably in adaptation metrics, improvements in walking and fatigue were largely retained, supporting the notion that short-term EMS can produce medium-term functional gains even after treatment cessation.

The improvements observed in this study are likely attributable to several interconnected neurophysiological mechanisms. One contributing factor may be reciprocal inhibition, where stimulation of antagonist muscles, such as the hamstrings or tibialis anterior, leads to suppression of overactive agonists like the quadriceps or gastrocnemius, thereby reducing spasticity (38). Another important mechanism involves afferent activation, as low-frequency electrical stimulation enhances somatosensory input, facilitating proprioceptive integration and potentially improving balance (39). In addition, neuroplasticity may be promoted through repeated peripheral stimulation, which can modulate cortical excitability and support synaptic reorganization (40). Muscle activation also plays a role, as even sub-threshold stimulation can help preserve baseline muscle tone and prevent disuse-related atrophy, which is particularly relevant in MS (41). Finally, autonomic effects resulting from low-level generalized stimulation may influence the balance between sympathetic and parasympathetic activity, contributing to the observed improvements in fatigue (42). These mechanisms act synergistically to promote motor relearning and improved postural control, especially when stimulation is paired with light activity, as in this study. However, since direct physiological measures were not included, these interpretations should be considered as hypotheses rather than demonstrated mechanisms. Further studies, including instrumental evaluations, are indeed necessary to confirm this interpretation.

Existing literature supports the use of TENS for reducing spasticity and improving function in MS. TENS has been shown to decrease muscle tone and spasm frequency in patients with neurological disorders (20, 22, 25).

The EMS builds upon this evidence by offering a full-body, wearable stimulation system that targets multiple muscle groups simultaneously, improving balance and mobility in patients with MS, cerebral palsy, and stroke. Our study confirmed these effects and further demonstrated improvement in fatigue and trunk control. Unlike traditional TENS units that target localized areas, the EMS enables systemic neuromodulation and can be used in conjunction with daily activities, potentially enhancing its rehabilitative impact.

Moreover, the use of robotic assessment platforms like Hunova is supported by research validating their sensitivity in detecting subtle changes in balance and coordination (37). The use of Hunova platform allowed quantification of motor responses that would likely be missed through observational or clinical scales alone.

The implementation of EMS in clinical practice offers several important advantages that support its potential as a complementary rehabilitation tool. As a home-based intervention, EMS facilitates access to therapy for individuals with limited mobility and may help reduce the burden on healthcare systems by enabling decentralized care. One of its notable strengths lies in the ability to personalize stimulation protocols: electrode placement and parameters can be tailored to address each patient's specific motor deficits and symptom profiles, thereby optimizing therapeutic outcomes.

The device's design, being wearable, non-invasive, and user-friendly, also supports high levels of adherence. In this case, the patient demonstrated full compliance throughout the study period, underscoring the acceptability and feasibility of regular EMS use in a home setting. Additionally, the intervention was well tolerated, with no adverse events reported, consistent with existing literature indicating a favorable safety profile.

Given the complexity of managing spasticity and fatigue in individuals with MS, particularly in those who are not candidates for pharmacological or invasive therapies, EMS represents a promising adjunct to conventional rehabilitation strategies. It may provide a non-invasive alternative to enhance motor function, potentially delaying the need for more intensive or less accessible interventions.

This study is subject to several limitations that should be taken into consideration. Primarily, its single-case design inherently restricts the generalizability of the findings. Although the within-subject observations offer detailed insight into the patient's response, they do not allow for extrapolation across different MS phenotypes or varying degrees of disability. Furthermore, the absence of a control or sham-stimulation group introduces the possibility of placebo effects, limiting the strength of causal inferences. While the use of the Hunova platform provides objective and quantifiable data, incorporating a control condition would enhance the methodological rigor and help isolate the specific effects of the EMS intervention.

Additionally, all the assessments were evaluated by a single evaluator who was aware of the intervention timeline, rising possible measurement bias. It is worth highlighting that the study design requires that the evaluation has to be performed by a single clinician, and, in order to minimize this bias, he/she adhered to standardized instructions and scoring protocols for all clinical tests (TUG, 6MWT, 10MWT). Furthermore, assessments were performed at the same time of day to minimize the confounding influence of daily fluctuations in MS symptoms, particularly fatigue. Regarding the Hunova-based evaluation, while the Hunova system provides objective biomechanical data, it is possible that assessor interaction can still influence the evaluation. However, the system's advantage lies in its ability to automatically calculate parameters through integrated sensors, reducing manual scoring error. Notably, widely accepted MCIDs for specific Hunova parameters (e.g., CoP ellipse area or stabilization time) are not yet established in the literature. Another notable limitation is the lack of neurophysiological assessments, such as electromyography (EMG) or transcranial magnetic stimulation (TMS), which could have offered direct evidence of changes in muscle recruitment patterns or corticospinal excitability resulting from the stimulation (43–45). Moreover, although the inclusion of a 1-Month follow-up period provides some insight into the sustainability of treatment effects, the long-term durability of the observed improvements remains unclear. It is not yet known whether these functional gains persist over longer durations (e.g., three or six months), or whether maintenance sessions may be necessary to preserve benefits.

Future research should aim to address these limitations by conducting larger, randomized controlled trials to establish efficacy and define optimal stimulation protocols. Stratifying participants by MS subtype, lesion distribution, and degree of spasticity may help identify the patient profiles most likely to benefit from EMS. Additionally, studies combining EMS with conventional physiotherapy or balance training could clarify potential synergistic effects. The inclusion of objective biomarkers, such as EMG, electroencephalography (EEG), functional near infrared spectroscopy (fNIRS) or magnetic resonance imaging (MRI), would further elucidate the neurophysiological mechanisms underlying functional changes (46–49). Advances in wearable technology could also facilitate remote tracking of usage patterns and response, enabling dose optimization and individualized therapy. Lastly, evaluations of cost-effectiveness will be essential to determine the feasibility of integrating EMS into standard neurorehabilitation pathways.

Collectively, such research will be critical to validating the broader clinical utility of EMS not only in MS, but also in other neurological conditions characterized by motor impairment, including stroke, spinal cord injury, and cerebral palsy.

## 5 Conclusions

This case study adds to the growing body of evidence supporting the EMS as a viable, non-invasive neuromodulation tool for improving motor function in MS. Over one month of use, the patient exhibited meaningful improvements in walking, balance, and fatigue, benefits that were sustained even after stimulation stopped. The integration of robotic assessments provided robust, objective evidence of postural and neuromuscular adaptation. While more extensive trials are needed, these findings underscore the promise of wearable EMS systems in expanding the toolkit for home-based neurorehabilitation and empowering individuals with chronic neurological conditions to reclaim function and independence.

## Data Availability

The raw data supporting the conclusions of this article will be made available by the authors, without undue reservation.
